# Rectus sheath hematoma in patients receiving subcutaneous enoxaparin: A case series of five patients

**DOI:** 10.1002/ccr3.3427

**Published:** 2020-10-27

**Authors:** Diogo Mendes, Ana Penedones, Michele Martins, Susana Cavadas, Carlos Alves, Francisco Batel‐Marques

**Affiliations:** ^1^ UFC – Coimbra Regional Pharmacovigilance Unit CHAD – Centre for Health Technology Assessment and Drug Research AIBILI – Association for Innovation and Biomedical Research on Light and Image Coimbra Portugal; ^2^ DruSER.Net – Drug Safety and Effectiveness Research Network Coimbra Portugal; ^3^ CHBV – Centro Hospitalar do Baixo Vouga Aveiro Portugal; ^4^ Laboratory of Social Pharmacy and Public Health School of Pharmacy University of Coimbra Coimbra Portugal

**Keywords:** drug‐related side effects and adverse reactions, enoxaparin, hematoma, heparin, low‐molecular‐weight, pharmacovigilance, rectus abdominis

## Abstract

Physicians must acknowledge the potential risk of RSH with enoxaparin. Switching home anticoagulation by enoxaparin upon hospital admission is common, but it may put patients at higher risk for RSH. Management guidelines are needed in this setting.

## Graphical abstract

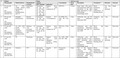

## 1 INTRODUCTION

Rectus sheath hematoma (RSH) is a potentially life‐threatenting bleeding complication which may result from several causes, including anticoagulation therapy. This paper reports five cases of RSH (one fatal) probably induced by subcutaneous enoxaparin that were confirmed by abdominal ultrasounds and/or computerized tomographic (CT) scans in a single hospital within 4 months.

Rectus sheath hematoma (RSH) may be caused by the rupture of epigastric arteries or the rectus muscle itself within the rectus sheath.^1^ It is a relatively rare clinical condition which accounts for less than 2% of patients with acute abdominal pain.^2^ Risk factors for RSH include anticoagulation therapy (ACT), stage ≥3 chronic kidney disease (CKD), abdominal wall injections, steroid or immunosuppressant therapy, abdominal trauma or surgery, cough, femoral puncture, and antiplatelet therapy (APT).^3^ Ultrasonography of the abdominal wall is a useful test to identify RSH. However, computerized tomographic (CT) scan should be carried out instead, since it is more sensitive and specific, being the standard diagnostic procedure.^2^, ^4^, ^5^ The diagnosis should be established as earlier as possible because, although its course is usually benign, RSH can also lead to serious adverse clinical outcomes, including death.^3^, ^6^, ^7^, ^8^, ^9^ The mortality rate associated with RSH was estimated around 2%‐4% overall,^6^, ^10^ though it may be as high as 25% among patients receiving ACT.^10^

During the last years, low‐molecular‐weight heparin (LMWH) has been routinely administered to hospitalized patients as prophylaxis of deep vein thrombosis (DVT).^11^ Furthermore, therapeutic dosing is a common practice to manage prevalent diagnoses, such as pulmonary embolism (PE), acute DVT, acute coronary syndrome (ACS), and atrial fibrillation (AF).^9^ Enoxaparin is a LMWH that enhances the activity of anti‐thrombin III (ATIII), which increases the inhibition of factor Xa (and other coagulation factors) and consequently downregulates coagulation.^12^ LMWH therapy can be associated with hemorrhagic adverse events, such as gastrointestinal bleeding.^13^ Although rare, cases of RSH have also been observed in patients treated with LMWH.^3^, ^6^, ^7^, ^14^, ^15^

This article presents five cases of RSH probably associated with subcutaneous injections of enoxaparin that were diagnosed in a single hospital within a 4‐month period of time.

## 2 METHODS

We retrospectively analyzed the characteristics of patients receiving subcutaneous injections of enoxaparin who were diagnosed with RSH at “Centro Hospitalar do Baixo Vouga” (Aveiro, Portugal) from November 2017 to February 2018. Medical records were reviewed to identify demographic characteristics (age, gender), clinical history, and cause of hospitalization, as well as home and hospital ACT (dose schedules and therapeutic indications), concomitant medication, symptoms, laboratory tests, and imaging procedures supporting the diagnosis, and treatment, duration, and outcome of RSH. Only RSH diagnoses confirmed by abdominal ultrasound and/or CT scan were considered. The cases were reported to the Regional Unit of Coimbra of the Portuguese Pharmacovigilance System (PPS). The Guidelines for Submitting Adverse Event Reports for Publication were followed to report study results.^16^

## 3 RESULTS

### 3.1 Case series

The five cases are described below. A summary of the main characteristics of each case is presented in Table 1. Three patients were female. The age of patients ranged between 67‐85 years old. Before hospitalization, three patients were on ACT at home (warfarin, n = 2; dabigatran etexilate, n = 1) because of underlying AF. The reasons for hospitalization included respiratory infection (n = 3), DVT (n = 1), and respiratory failure (n = 1). The time interval between initiation of subcutaneous enoxaparin and RSH onset ranged between 2 and 12 days. Two cases of RSH were confirmed with abdominal ultrasound, two with CT scan and one with both imaging procedures. One patient died because of RSH. One patient recovered from the RSH but died later because of hospital‐acquired pneumonia. Three patients recovered in full.

**Table 1:** Characteristics of the cases of rectus sheath hematoma (RSH) induced by subcutaneous enoxaparin

#	Patient			Drugs			Adverse event				
	Demography	Medical history	Hospitalization reason	Suspect		Concomitant	Onset time^a^	Description	Treatment^b^	Duration	Outcome
				Dose schedule	Indication						
1	85‐y‐old, female (PT‐INFARMED‐B201805‐241)	AF, CHF, T2DM, arterial hypertension, obesity, hypothyroidism, and CVI	Respiratory failure	Enoxaparin, 80 mg, qd, SC, for 8 d, 80 mg, bid, SC, for 6 d, 80 mg, qd, SC, afterward	Replace/home ACT (oral warfarin) for AF	MTP (IV)	12 d	Renal injury with anuria and hypotension; RSH and hematoma on the right thigh; abdominal CT scan: hemorrhage; hemorrhagic shock	Blood transfusion, surgery/embolization of bleeding arteries	Few days (not specified)	Death
2	80‐y‐old, female (PT‐INFARMED‐B201805‐262)	Idiopathic bronchiectasis	Infected bronchiectasis	Enoxaparin, 40 mg, qd, SC, for 5 d	Hospital ACT: VTE prophylaxis	O_2_, PRDL (IV), NAC, IB, CAZ, CIP	5 d	Abdominal pain and Hb drop; abdominal ultrasound: RSH	Analgesics and local ice	1 d	Recovered
3	81‐y‐old, male; (PT‐INFARMED‐B201805‐940)	AF, CHF, CKD (eGFR < 15 mL/min), respiratory insufficiency, thrombocytopenia	Influenza B virus‐associated bronchopneumonia	Enoxaparin, 60 mg, qd, SC, for 5 d, 20 mg, qd, for 1 d	Replace/home ACT (oral warfarin) for AF	MTP (IV), CXM, OTV	2 d	Abdominal pain with tumefaction; abdominal ultrasounds and later CT scan: RSH	Blood and plasma transfusion	9 d	Recovered
4	67‐y‐old, male (PT‐INFARMED‐B201806‐781)	AF	Pneumonia	Enoxaparin, 80 mg, bid, SC, for 4 d	Replace/home ACT (oral dabigatran etexilate, 110 mg, bid) for AF	O_2_, AZM, AMC, SXT, IB + SBL	4 d	Abdominal pain and nausea; Hb drop; CT scan: RSH; abdominal aortic thrombus; abdominal aortic aneurysm enlargement; hemorrhagic shock	Blood transfusion; embolization of bleeding arteries	Few days (not specified)	Recovered^c^
5	72‐y‐old, female (PT‐INFARMED‐B201905‐992)	Unknown	DVT	Enoxaparin, 60 mg, bid, SC, for 8 d	Hospital ACT: DVT	Fluconazole, warfarin	8 d	Pain in the inguinal region, with further worsening and involvement of iliac fossa, tumefaction; abdominal ultrasound: RSH and peritoneal bleeding	Blood transfusion; embolization of bleeding arteries	4 d	Recovered

Abbreviations: AAS, acetylsalicylic acid; ACE‐i, angiotensin converting enzyme inhibitor; ACT, anticoagulant therapy; AF, atrial fibrillation; AMC, amoxicillin‐clavulanic acid; AZM, azithromycin; bid: twice daily; CAZ, ceftazidime; CHF, Congestive heart failure; CIP, ciprofloxacin; CKD, Chronic kidney disease; CVI, chronic venous insufficiency; CXM, cefuroxime; DVT, deep vein thrombosis; eGFR, estimated glomerular filtration rate; Hb, hemoglobin; IB, ipratropium bromide; IV, intravenous; MTP, methylprednisolone; NAC, N‐acetylcysteine; O_2_, oxygen; OTV, oseltamivir; PRDL, prednisolone; qd, once daily; RSH, rectus sheath hematoma; SBL, salbutamol; SC, subcutaneous injection; SXT, trimethoprim‐sulfamethoxazole; T2DM, Type 2 diabetes mellitus; VTE, venous thromboembolism.

^a^ Onset time: time elapsed between the start of therapy with enoxaparin sodium and the onset of the adverse event.

^b^ Treatment included discontinuation of enoxaparin.

^c^ This patient (case 4) recovered of the RSH but died 13 d later because of hospital‐acquired pneumonia.

#### 3.1.1 Case 1

**FIGURE 1:**
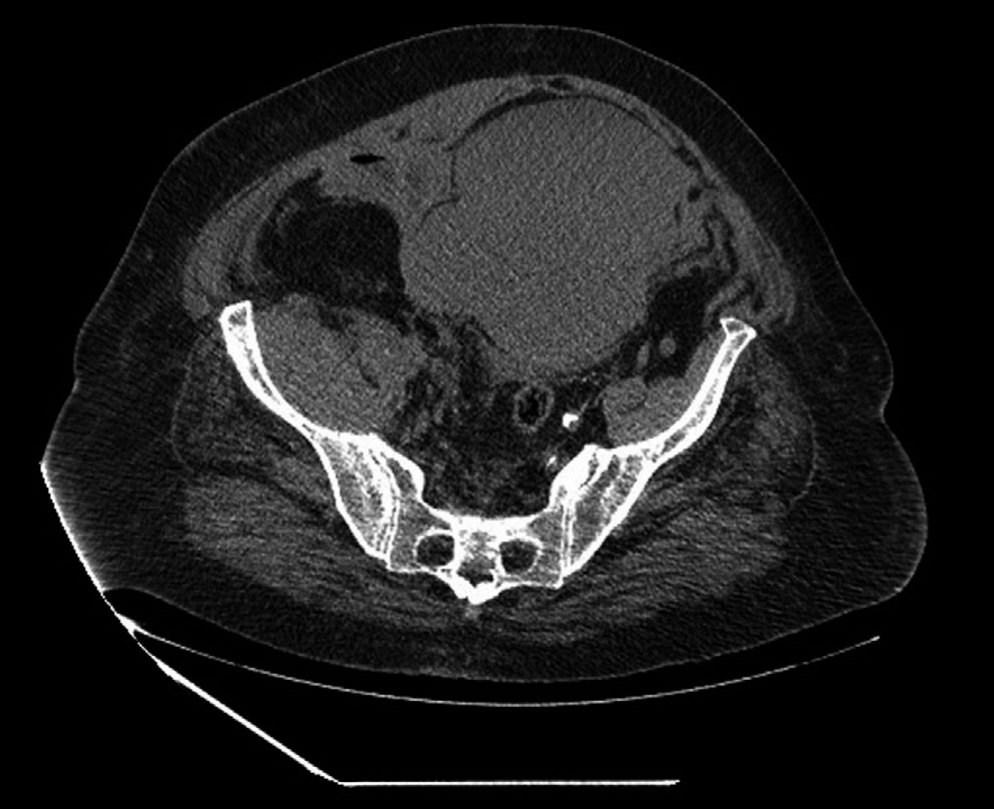
Case 1: CT scan of the abdomen and pelvis revealing a rectus sheath hematoma. Two relatively communicating high‐density expansive formations, the largest, anterior to the bladder, and the other, slightly lower and lateralized to the right, extending to the meso rectum, compatible with two collected hematomas

A 85‐year‐old female patient was admitted to hospital due to respiratory failure. Her clinical history included AF, congestive heart failure (CHF), type 2 diabetes mellitus (T2DM), arterial hypertension, obesity, hypothyroidism, and venous insufficiency of the lower limbs. The therapy included the initiation of IV methylprednisolone and the replacement of home ACT (warfarin used to treat AF) by enoxaparin (with dose adjustment for the renal function): 80 mg, SC, qd, during 8 days, followed by a dose increase to 80 mg, bid, for 6 days, and a further dose reduction to 80 mg, SC, qd, until the day of discharge to another hospital. Twelve days after initiation of therapy with enoxaparin, a phlebotomy was performed to extract 200 cc of blood due to high hemoglobin (Hb) count (19.8 g/dL), and the subsequent arterial blood gas (ABG) analysis showed Hb = 16.3 g/dL. On the following day, the patient presented Hb = 12.5 g/dL, hypotension, acute renal failure, and hyperlactacidaemia; she also had large hematomas on both the abdominal wall and the right thigh. An abdominal CT scan showed an active hemorrhage, and RSH was diagnosed (Figure 1). Hypotension was nonresponsive to fluid resuscitation (systolic blood pressure: 60 mm Hg). The patient progressed to hemorrhagic shock. Blood transfusions were performed, and she was further transferred to the department of surgery of another hospital for embolization of bleeding arteries. The patient died a few days later (PT‐INFARMED‐B201805‐241).

#### 3.1.2 Case 2

**FIGURE 2:**
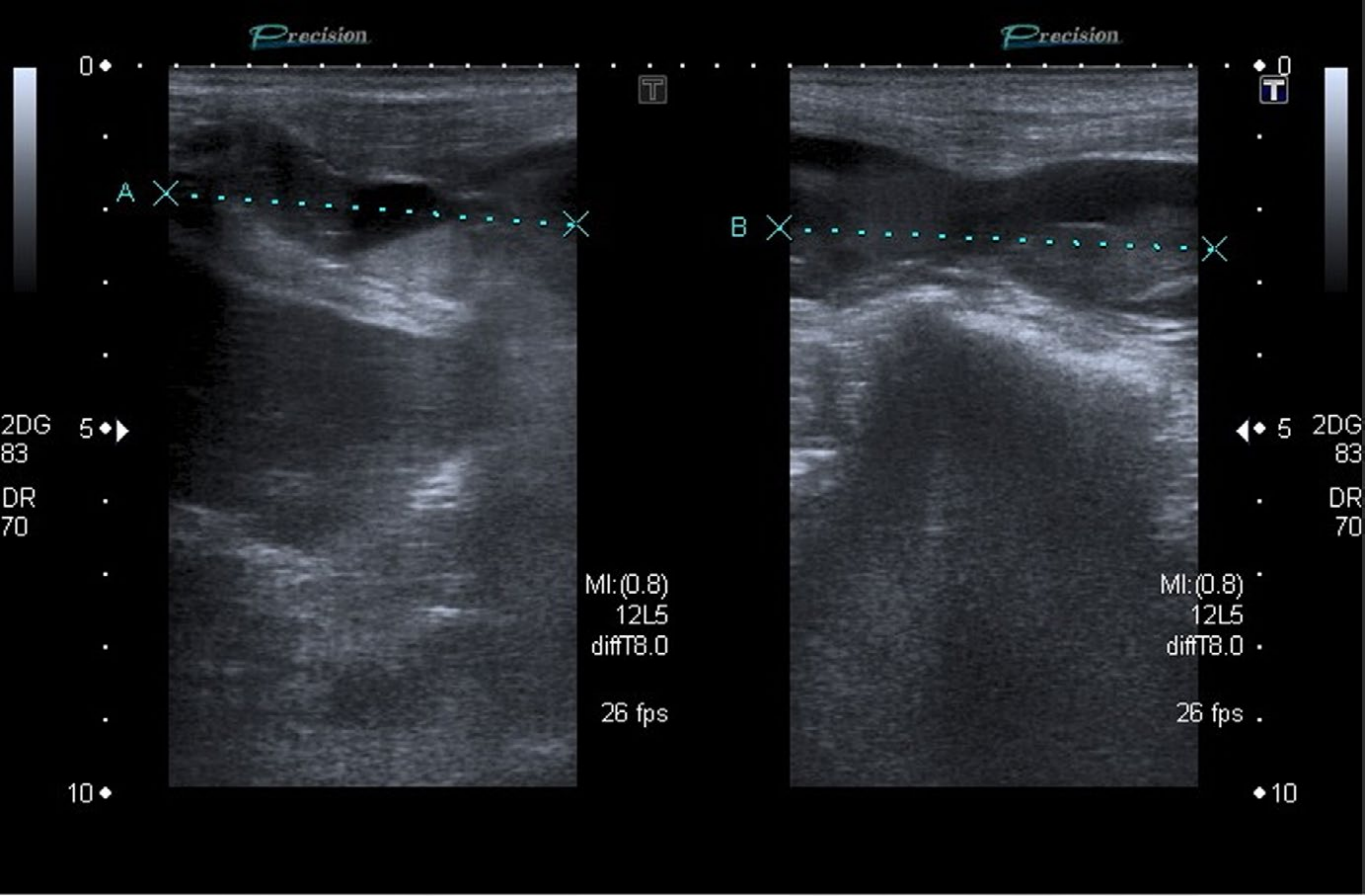
Case 2: Ultrasound of the abdomen revealing a right‐sided rectus sheath hematoma. Diffuse wall thickening from the upper to the lower insertion of the rectus abdominis muscle, with several internal liquid loci, within which solid material is observed, outlining the solid/liquid level, which is compatible with a hematoma (probably spontaneous) in the ultrasound image

A 80‐year‐old female patient was admitted to hospital due to infected bronchiectasis. Treatment with oxygen (O2), acetylcysteine, ipratropium bromide, antibiotics, and corticosteroids (oral prednisolone, 20 mg, qd) was initiated. Enoxaparin, 40 mg, SC, qd, was prescribed to prevent venous thromboembolism (VTE). Five days later, the patient presented abdominal pain and a large palpable mass in the right hypochondrium and flank. There was no hemodynamic rebound, but there was a drop of 1 g/L in the levels of Hb. An abdominal ultrasound showed a right‐sided RSH (Figure 2)—diffuse wall thickening from the upper to the lower insertion of the rectus abdominis muscle, with several internal liquid loci, within which solid material was observed, and with “fresh” and organized areas. The approach was based on conservative measures that included discontinuation of enoxaparin, analgesics, and ice packs. The patient recovered within 1 day (PT‐INFARMED‐B201805‐262).

#### 3.1.3 Case 3

**FIGURE 3:**
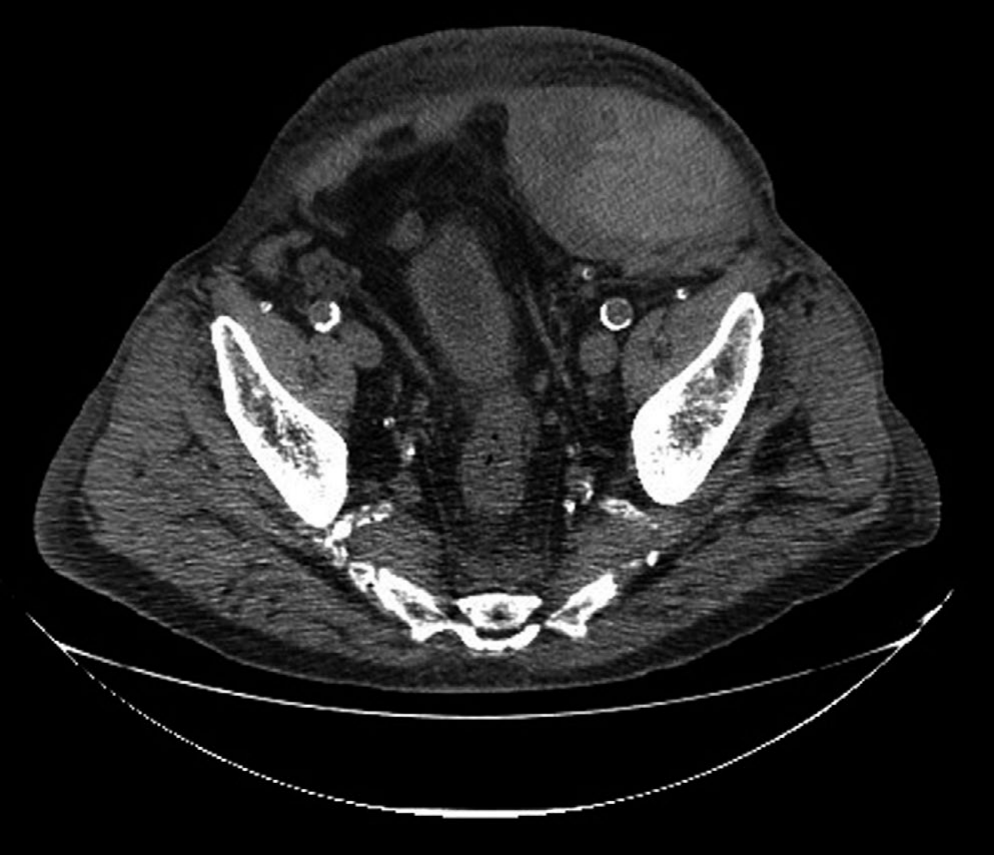
Case 3: CT scan of the abdomen and pelvis revealing a left rectus sheath hematoma. Heterogeneous thickening of the left large rectus abdominis muscle, with dense areas, revealing a hematoma with still relatively recent hemorrhage

A 81‐year‐old male patient was admitted to hospital due to Influenza B virus‐associated bronchopneumonia. The clinical history of the patient included CHF (biological prosthesis for aortic valve), CKD (estimated glomerular filtration rate (eGFR) <15 mL/min), type I respiratory failure, AF, and thrombocytopenia. Treatment with cefuroxime, oseltamivir, and IV methylprednisolone was initiated. Enoxaparin, 60 mg, SC, qd, for 5 days and 20 mg, SC, qd, for 1 day, was given to replace home ACT (oral warfarin) to prevent thromboembolism in AF. Two days later, the patient presented a fixed, nonreducible abdominal wall swelling, without hemodynamic rebound. The ultrasound showed a left‐sided RSH (95 × 48 × 92 mm) (Figure 3). A conservative clinical approach was adopted, including discontinuation of enoxaparin, blood and plasma transfusion and vigilance. A CT scan showed that the hematoma was sustained by the aponeurosis in the lower extremity of the left rectus abdominis. The rate of decrease in the level of Hb was slow, and therefore, conservative treatment was maintained. The patient recovered and was discharged 9 days later (PT‐INFARMED‐B201805‐940).

#### 3.1.4 Case 4

**FIGURE 4:**
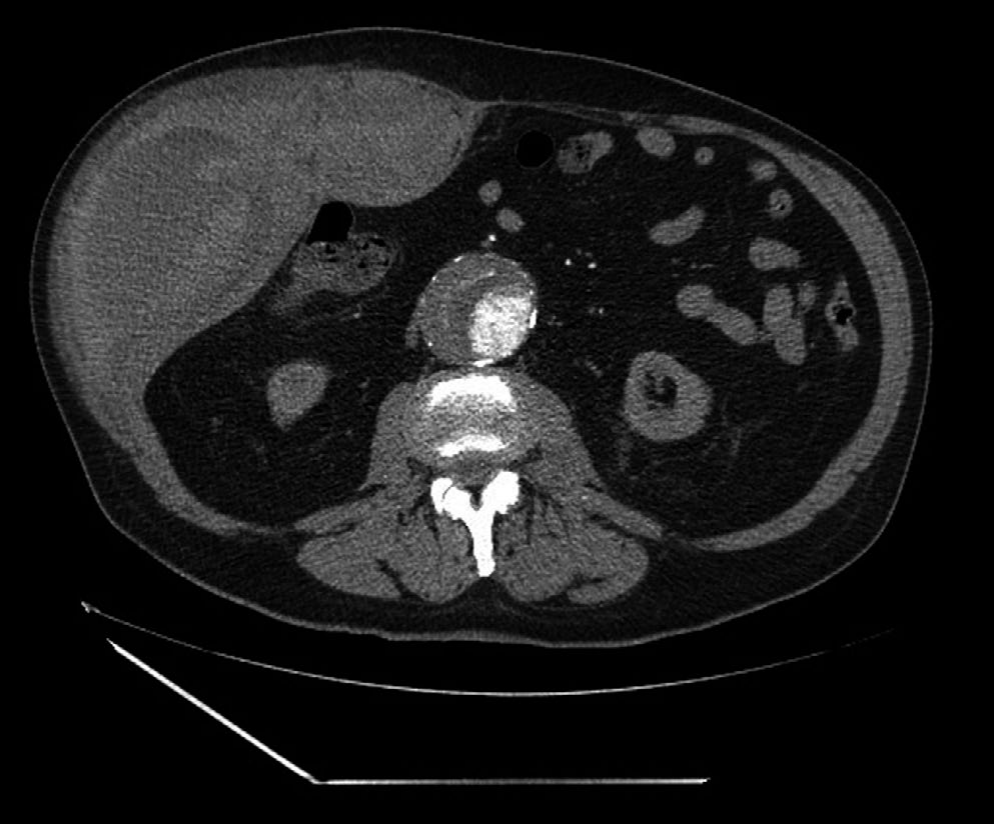
Case 4: CT scan of the abdomen and pelvis revealing a right rectus sheath hematoma. Massive nodular formation (heterogeneous), with solid and liquid areas, compatible with large hematoma depending on the anterior abdominal wall and right side

A 67‐year‐old male patient was admitted to hospital due to pneumonia. Treatment with O2, antibiotics, and salbutamol + ipratropium bromide was initiated. Enoxaparin, 80 mg, SC, bid, was given to replace home ACT (oral dabigatran etexilate, 110 mg, bid) due to AF. Four days later, the patient presented abdominal pain in the right hypochondrium, nausea without vomiting, and a large palpable mass in the right quadrants. There was a drop in the levels of Hb of 4 g/L. A CT scan showed a large hematoma (19 × 15 × 8 cm) with small foci of active bleeding in the upper portion, aneurysmal dilatation of the abdominal aorta with a maximum caliber of 58 mm over a length of 77 mm, and a parietal thrombus (maximum 25 mm) (Figure 4). Despite treatment, the patient progressed to hemorrhagic shock. Embolization of bleeding arteries and transfusion of 2 units of packed red blood cells were performed, two suction drains were placed in the hematoma cavities, and the patient was transferred to the intensive care unit (ICU). The patient initially recovered, but later developed hospital‐acquired pneumonia associated with invasive intubation/ ventilation and died 13 days later (PT‐INFARMED‐B201806‐781).

#### 3.1.5 Case 5

**FIGURE 5:**
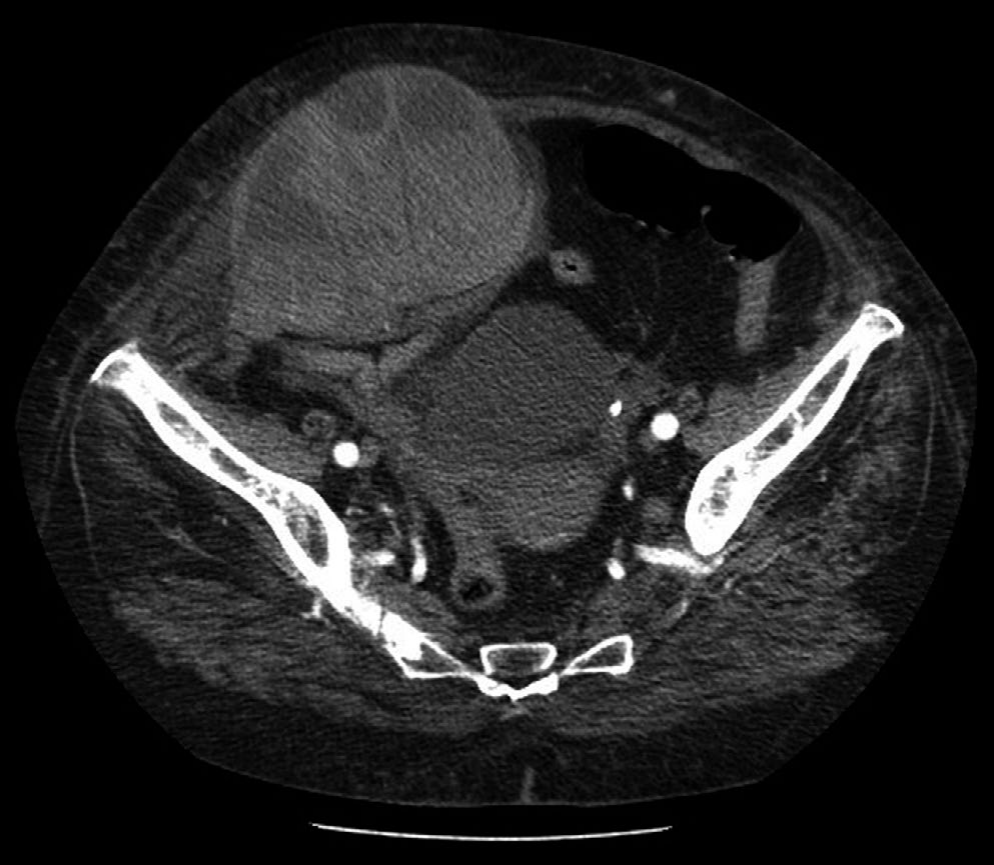
Case 5: CT scan of the abdomen and pelvis revealing a right rectus sheath hematoma. Large expansive oval formation depending on the abdominal wall of the right hemi‐abdomen, very markedly heterogeneous, with areas of greater and lesser density, compatible with a hematoma. After contrast injection (arterial phase), denser foci are observed, indicating active bleeding

A 72‐year‐old female patient was admitted to hospital due to DVT. Treatment with enoxaparin, 60 mg, SC, bid, was initiated. The patient was also on oral fluconazole (100 mg, qd) and 5 days later initiated warfarin (2.5 mg, qd). Eight days after having initiated enoxaparin, the patient had pain in the inguinal region, with further worsening, involving the iliac fossa and swelling. She was hemodynamically stable, but there was a decrease of 2 g/L in the levels of Hb and the international normalized ratio (INR) was estimated at 1.7. The abdominal ultrasound showed RSH (194 × 119 × 102 mm), later confirmed by CT scan (Figure 5). After contrast injection, denser intraluminal foci were observed, indicating active bleeding, but it was not possible to identify the bleeding vessel. There was also a marked densification of the peripheral muscle planes, probably due to hematic infiltration and surrounding effusion, and a small intraperitoneal effusion in the pelvic and sub‐hepatic cavity. Blood transfusion was performed. The patient was transferred to another hospital to perform embolization and recovered within 4 days (PT‐INFARMED‐B201905‐992).

## 4 DISCUSSION

This study reports a series of five cases of RSH probably induced by subcutaneous injections of enoxaparin, of which one was fatal. All RSH diagnoses were confirmed with either abdominal ultrasound or CT scan. Although a rare complication, these cases occurred in a single hospital over only 4 months. This led to changes in clinical practices, systems review, and training of healthcare personnel to prevent further episodes.

Studies have found that the incidence of RSH is higher in the female and elderly population.^3^, ^6^ The anatomic differences in rectus muscles between the two genders, as well as weakening of abdominal wall muscles because of stretching during pregnancy, predispose the female gender to a higher risk of RSH.^3^, ^6^, ^17^ Further, there is a progressive weakening of abdominal wall muscles with age.^18^

Several other risk factors for RSH have been identified in other studies. According to the evaluation of 115 patients hospitalized with RSH (confirmed by CT scan) in the United States, 77% of the individuals were on the ACT, 58% had CKD stage ≥3, 51% were receiving abdominal injections, and 42% were on steroids or immunosuppressant therapy; nearly 29% of the patients receiving subcutaneous abdominal injections were under LMWH therapy.^3^ In our study, we found that three patients (60%) were on steroids therapy. Whether the use of steroids increases the risk of RSH in patients under treatment with enoxaparin should be further investigated, since this association is frequently used in clinical practice and the evidence on this topic is scarce in both clinical literature and product monographs. As an example, the European Summary of Product Characteristics (SmPC) of the originator enoxaparin only mentions that “systemic glucocorticoids” “may be administered with caution concomitantly with enoxaparin sodium”.^12^

The use of a LMWH over a direct oral anticoagulants (DOAC) for VTE prophylaxis in acutely ill hospitalized medical patients is recommended in clinical practice guidelines.^19^ According to the results of a systematic review of three randomized controlled trials (RCTs) of low risk of bias, the use of DOACs increased the risk of major bleeding (Relative Risk [RR] = 1.99; 95% Confidence Interval [CI], 1.08‐3.65) but did not reduce the risk of symptomatic VTE versus the use of LMWHs in patients hospitalized because of acute diseases.^20^ However, most patients of the present case series (n = 3/5; 60%) were prescribed with enoxaparin upon the hospitalization to replace the ACT (warfarin or DOAC) they were already using to treat AF. Whether switching therapy from home ACT to subcutaneous LMWH is necessary deserves further research, particularly in the case of patients on ACT for other reasons than VTE prophylaxis. In the BRIDGE Trial, 1884 patients with AF were randomly assigned to receive bridging anticoagulation with LMWH or placebo after perioperative interruption of warfarin therapy.^21^ There was no difference in the incidence of arterial thromboembolism between the group of patients on LMWH bridging and the no‐bridging group (ie, without anticoagulation); however, the risk of major bleeding was higher in patients receiving LMWH bridging than in those with no‐bridging anticoagulation (RR = 2.44; 95% CI, 1.28‐5.0).^21^ Furthermore, the PAUSE cohort study reported low rates of major bleeding and arterial thromboembolism in 3007 patients with AF who needed to interrupt long‐term treatment with DOACs (without heparin bridging) before elective surgery or procedure.^22^ Therefore, perioperative bridging with LMWH may cause more harm than benefit in most anticoagulated patients with AF.^21^, ^22^, ^23^ Nevertheless, evidence is still lacking for patients with AF who are hospitalized because of acute illnesses or other reasons than surgical/invasive procedures. Further research needs to be carried out.

Physicians should be aware of clinical and imaging features of RSH, aiming at early diagnosis and conservative management approaches to prevent complications. Such awareness is important to preclude misdiagnosis and avoid inappropriate invasive procedures, given that symptoms and signs of RSH (ie, abdominal mass or pain, drop in hematocrit or hypotension, nausea, and vomiting) can mimic other conditions (eg, diverticulitis, appendicitis, and acute pancreatitis).^3^, ^9^, ^14^ Other signs and symptoms of RSH include abdominal distension or ecchymosis, peritoneal irritation, scrotal swelling, and tachycardia.^3^, ^6^ Physical examination procedures to differentiate intra‐abdominal from abdominal wall pathology were described elsewhere.^24^ The maneuver described by Carnett entails palpation of the tender abdomen in the supine and half‐sitting positions. While intra‐abdominal processes are most tender in the supine position and protected by the contracted rectus muscle when the patient is sitting, abdominal wall processes remain tender in both positions.^24^ Ultrasound and CT scan are the two main imaging procedures to diagnose RSH. Ultrasound can provide results in a faster way, but CT is 100% sensitive and specific and allows to understand whether there is active bleeding.^2^, ^9^, ^25^ Most RSH diagnosis (n = 3/5; 60%) were confirmed by CT in our study. The use of these imaging procedures allows to confirm RSH diagnoses in a heterogeneous population of patients with varying demographic and clinical characteristics. According to Berná et al, RSH is classified into three types based on CT results—type I: intramuscular, unilateral hematoma which does not dissect into the fascial plane; type II: intramuscular (though with blood between the muscle and the transversalis fascia), unilateral or (usually) bilateral hematoma, with no blood occupying the prevesical space; and type III: with or without muscle involvement and blood can be seen within the transversalis fascia, peritoneum, and prevesical space.^26^ While the treatment of type I and type II RSH is usually conservative, type III RSH often requires blood transfusion.^26^ If type III RSH leads to hemodynamic instability that is uncontrollable with fresh frozen plasma and fluid resuscitation, invasive treatment may be required; there are two options: angiography with embolization of the bleeding vessel; or surgery with hematoma evacuation, ligation of bleeding vessels, and closed‐suction drainage. The success rate of invasive therapy is usually high in such cases.^8^, ^27^ Only one out of the five cases reported was successfully managed with conservative approaches. Angiography with embolization of the bleeding vessel was performed for three patients, of which one died.

This study presents some limitations, mainly due to the lack of information that was not possible to retrieve in some cases. According to the clinicians who reported the cases, the administration of enoxaparin was initiated on therapeutic doses (with adjustment to the renal function of each patient) and at least 24 hours after the last intake of an anticoagulant for those on ACT at home. Since we are not aware of the full clinical condition of each patient, it is not possible to ascertain whether dose schedules were properly adjusted. It is possible that the clinical history and the concomitant medication were provided in more detail for some patients rather than others, as the information was directly collected from the reporting physicians. Therefore, we were not able to assess the influence of these concomitant factors on the risk of RSH. Furthermore, two patients were transferred to departments of surgery of other hospitals, and therefore, it was not possible to describe in detail the treatment provided for such patients.

## 5 CONCLUSION

Physicians must be aware of the potential risk of RSH induced by subcutaneous injections of enoxaparin, which is commonly prescribed to prevent thromboembolic events in hospitalized patients, even when used under therapeutic adjusted doses. Whether the use of systemic corticosteroids increases the risk of RSH in patients under treatment with subcutaneous enoxaparin deserves further investigation. Home anticoagulation therapies are frequently replaced by subcutaneous enoxaparin when patients are hospitalized, but this practice may put patients at higher risk of RSH. Therefore, updated guidelines on the management of home anticoagulation (ie, switching or maintaining therapy) in hospitalized patients are needed.

## CONFLICT OF INTEREST

None declared.

## AUTHOR CONTRIBUTIONS

DM and FBM: were involved in the conception and design. MM and SC: collected data. DM, AP, and CA: supervised the collection of data and analyzed the data. DM: wrote the first draft of the paper. DM, AP, SC, CA, and FBM: revised it critically for intellectual content. All authors made substantial contributions, approved the final version of the manuscript, and agreed to be accountable for all aspects of the work.

## ETHICAL APPROVAL

Ethical approval was not required, since data were retrieved from pseudononymized case reports already submitted to the Portuguese Pharmacovigilance System (PPS).
